# *Mythimna separata* herbivory primes maize resistance in systemic leaves

**DOI:** 10.1093/jxb/erab083

**Published:** 2021-02-27

**Authors:** Saif ul Malook, Yuxing Xu, Jinfeng Qi, Jing Li, Lei Wang, Jianqiang Wu

**Affiliations:** 1 Department of Economic Plants and Biotechnology, Yunnan Key Laboratory for Wild Plant Resources, Kunming Institute of Botany, Chinese Academy of Sciences, Kunming 650201, China; 2 CAS Center for Excellence in Biotic Interactions, University of Chinese Academy of Sciences, Beijing 100049, China; 3 The James Hutton Institute, UK

**Keywords:** Benzoxazinoids, insect resistance, jasmonic acid, maize, *Mythimna separata*, priming, transcriptome

## Abstract

Biotic and abiotic cues can trigger priming in plants, which enables plants to respond to subsequent challenge with stronger and/or faster responses. It is well known that herbivory activates defense-related responses in systemic leaves. However, little is known about whether insect feeding activates priming in systemic leaves. To determine whether and how herbivory induces priming in maize systemic leaves, a combination of insect bioassays, phytohormone and defense metabolite quantification, and genetic and transcriptome analyses were performed. Actual and simulated *Mythimna separata* herbivory in maize local leaves primed the systemic leaves for enhanced accumulation of jasmonic acid and benzoxazinoids and increased resistance to *M. separata*. Activation of priming in maize systemic leaves depends on both the duration of simulated herbivory and perception of *M. separata* oral secretions in the local leaves, and genetic analysis indicated that jasmonic acid and benzoxazinoids mediate the primed defenses in systemic leaves. Consistently, in response to simulated herbivory, the primed systemic leaves exhibited a large number of genes that were uniquely regulated or showed further up- or down-regulation compared with the non-primed systemic leaves. This study provides new insight into the regulation and ecological function of priming in maize.

## Teaser

*Mythimna separata* herbivory primes systemic leaves, and this priming-endowed resistance requires perception of insect oral secretions, the plant hormone jasmonic acid, and the defensive metabolites benzoxazinoids.

## Introduction

Plants are sessile organisms that are often challenged by various adverse environmental factors, including insect attack. They have evolved sophisticated defense mechanisms to fend off insect herbivores. Different plants are able to synthesize structurally diverse secondary metabolites, many of which are toxic, repellent, or anti-digestive for insects (Mithofer and Boland, 2012). Constitutive defenses are physical as well as chemical defense traits that are always present regardless of herbivory. Defenses are costly. Thus, plants often depend on inducible defenses to fight against insects, as they are displayed or enhanced only after herbivore attack, allowing plants to reserve energy and resources for growth and development under insect-free conditions (Erb and Reymond, 2019).

Maize (*Zea mays*) is one of the most important food crops and is cultivated around the globe with production of more than 1.14 billion tons in 2018 (http://www.fao.org/faostat/en/#data/QC/visualize). Maize often suffers from insect attack, such as from the chewing insects *Ostrinia nubilalis* and *Spodoptera frugiperda* and the piercing-sucking insect *Rhopalosiphum maidis*, resulting in large yield losses (Carena and Glogoza, 2004; Burtet *et al.*, 2017; Hassan *et al.*, 2018). The phytohormone jasmonic acid (JA) plays a central role in regulating defense of plants, including maize, against insects (Howe and Jander, 2008; Qi *et al.*, 2018; Erb and Reymond, 2019). Mechanical wounding rapidly induced highly increased JA in maize, and applying the oral secretions (OS) of the insect *Mythimna separata* to fresh mechanical wounds (to simulate insect feeding) induced levels of JA almost 2-fold higher than from mechanical wounding, indicating that maize is able to perceive certain elicitors in insect OS and deploy JA-dependent defenses (Qi *et al.*, 2016). The *LOX8* gene encodes a 13-lipoxygenase enzyme that is involved in JA biosynthesis in maize; beet armyworm (*Spodoptera exigua*) caterpillars fed on the *lox8/tasselseed1* maize mutants showed better growth compared with those fed on the wild-type (WT) plants (Tzin *et al.*, 2017). Maize mutants lacking *OPR7* and *OPR8*, which encode two 12-oxo-phytodienoate reductases, have remarkably reduced JA content, and beet armyworms consumed more tissues on the *opr7 opr8* mutant plants and grew larger than on the WT maize plants (Yan *et al.*, 2012). In addition to phytohormone signaling, large-scale transcriptomic and metabolic rearrangements are critical for maize deployment of defenses. RNA-seq analysis indicated that maize responds to simulated *M. separata* feeding with transcriptional regulation of a large number of genes (Qi *et al.*, 2016). Tzin *et al.*, (2015) found that the aphid *R. maidis* feeding on maize induced the strongest transcriptomic and metabolomic changes in the first few hours; however, after 4 d, both the transcriptomes and metabolomes of the aphid-infested maize became more similar to those of the non-aphid-infested maize. The maize inbred lines B73 and Mo17 are relatively susceptible and resistant to aphids, respectively, and these maize lines exhibited distinct transcriptional responses before and after the feeding from the aphid *Rhopalosiphum padi* (Song *et al.*, 2017).

Benzoxazinoids (Bxs) are secondary metabolites with the 2-hydroxy-2*H*-1,4-benzoxazin-3(4H)-one skeleton that are only found in some species of cereals, including maize (Wouters *et al.*, 2014, 2016). The Maize B73 genome encodes three indole-3-glycerolphosphate synthase enzymes, which catalyse the conversion of 1-(2-carboxyphenylamino)-l-deoxyribulose-5-phosphate to indole-3-glycerolphosphate (Wisecaver *et al.*, 2017; Richter *et al.*, 2021). A series of enzymes BX1 to BX9 and indole-3-glycerol phosphate lyase catalyse the conversion of indole-3-glycerolphosphate into 2,4-dihydroxy-7-methoxy-2*H*-1,4-benzoxazin-3-one glucoside (DIMBOA-Glc) (Frey *et al.*, 1997, 2009; Niemeyer, 2009). Recently, two enzymes, a 2-oxoglutarate-dependent dioxygenase (BX13) and an *O*-methyltransferase (BX14) in the Bx biosynthesis pathway were identified (Handrick *et al.*, 2016). BX13 catalyses the conversion of DIMBOA-Glc into 2,4,7-trihydroxy-8-methoxy-1,4-benzoxazin-3-one glucoside (TRIMBOA-Glc) and BX14 converts 2,4-dihydroxy-7,8-dimethoxy-1,4-benzoxazin-3-one glucoside (DIM_2_BOA-Glc) to 2-hydroxy-4,7,8-trimethoxy-1,4-benzoxazin-3-one glucoside (HDM_2_BOA-Glc) (Handrick *et al.*, 2016). Most of the maize Bxs are induced by insect feeding. DIMBOA-Glc and its methylation product HDMBOA-Glc accumulate in response to *Leucania separata* (rice armyworm) herbivory and the increase of these metabolites is associated with elevated resistance to *S. exigua* (Oikawa *et al.*, 2004; Tzin *et al.*, 2017). *Mythimna separata*, *Diabrotica virgifera virgifera*, and *Spodoptera littoralis* feeding all increased the levels of 2,4-dihydroxy-7-methoxy-1,4-benzoxazin-3-one (DIMBOA) in the damaged leaves of maize (Erb *et al.*, 2009; Maag *et al.*, 2016; Qi *et al.*, 2016). *Spodoptera littoralis* and *S. exigua* larvae show decreased growth when fed on an artificial diet containing DIMBOA (Rostas, 2007; Glauser *et al.*, 2011). The defensive functions of Bxs were also demonstrated by genetic studies. Compared with WT maize, maize mutant *igps1* has reduced resistance to *S. exigua* (Richter *et al.*, 2021), and the *bx1* and *bx2* mutants are also susceptible to the aphid *R. maidis* and *S. exigua* (Tzin *et al.*, 2015, 2017).

Studies in various species have indicated that plants respond to wounding or herbivory not only in the damaged leaves (local leaves) but also in the other undamaged leaves and even in roots (Heil and Ton, 2008; Howe *et al.*, 2018). In addition to the local insect-damaged leaves, maize systemic leaves also have strong defense-related responses. Wounding increased the expression levels of *allene oxide synthase* (*AOS*), transcription factor *MYC7*, and *ribosome inactivating protein* only at the treatment site, whereas *N*-linolenoyl-glutamine, an elicitor in the OS of *S. exigua*, strongly induced the expression of these genes throughout the whole maize leaf (Engelberth *et al.*, 2007, 2012). Simulated *M. separata* herbivory elicited accumulation of JA and Bxs as well as large transcriptomic changes in systemic unwounded maize leaves (Malook *et al.*, 2019).

After receiving stimuli such as pathogens, insects, chemical cues, or abiotic stresses some plants may enter a primed physiological state (this process is named priming), allowing plants to mount a faster and/or stronger defense response to subsequent challenges (Mauch-Mani *et al.*, 2017). Treating tomato (*Solanum lycopersicum*) seeds with JA or β-aminobutyric acid primed the tomato plants for increased defenses against *Manduca sexta*, *Myzus persicae*, and *Tetranychus urticae*, and the herbivore resistance was associated with increased expression levels of *allene oxide synthase 2*, *proteinase inhibitor II* and the pathogenesis-related gene *PR1b1* (Worrall *et al.*, 2012). Oviposition also induces priming in some plant species. For example, compared with oviposition-unexperienced *Nicotiana attenuata* plants, those oviposited by the *S. exigua* showed higher resistance to *S. exigua* larvae, as these insects had elevated mortality, retarded development, and inflicted less feeding damage on plants (Bandoly *et al.*, 2015). Beet armyworm (*S. exigua*) larvae exhibited reduced growth on *M. sexta*-oviposited *N. attenuata* plants, and similarly, *S. exigua*-oviposited plants had elevated resistance to *M. sexta* (Bandoly *et al.*, 2016). Herbivore-induced plant volatiles (HIPVs) can also function as priming agents. Maize seedlings previously exposed to green leaf volatiles from the neighboring maize plants had greater levels of JA and sesquiterpenes upon wounding or simulated *S. exigua* feeding than did the control non-exposed maize seedlings (Engelberth *et al.*, 2004). Erb *et al.* (2015) demonstrated that maize seedlings treateted with the OS from *S. littoralis* emitted indole, and indole exposure primed the production of phytohormones, green leaf volatiles, and mono- and homoterpenes in systemic leaves, and furthermore, herbivory-induced indole enhanced the induction of defensive volatiles in the neighbouring maize plants. *Diabrotica virgifera virgifera* infestation on roots of maize seedlings increased the DIMBOA contents in leaves, and the leaves were primed for accumulation of chlorogenic acid after subsequent infestation by *S. littoralis* (Erb *et al.*, 2009).

After initial insect feeding, the same insects may subsequently migrate to other leaves, and the systemic leaves could also be attacked by other insects of the same or different species. In this study, we investigated whether maize systemic leaves can be primed for enhanced resistance to insects and what the underlying mechanisms are. We show that insect feeding on maize seedlings primed the systemic leaves for enhanced insect resistance. Genetic and biochemical analysis indicated that JA signaling and Bx accumulation are required for the primed defenses. Moreover, the transcriptome indicated large transcriptional changes in these systemic leaves.

## Materials and methods

### Plant growth and oral secretion collection

The seeds of maize inbred lines A188, B73, W22, and the mutants *bx2::Ds* and *lox8/tasselseed1* were germinated in 12-cm-diameter plastic pots filled with commercial potting soil and vermiculite (about 7:1 ratio) under natural light conditions (about 12–14 h day length) in a greenhouse (25±4 °C day, 20±4 °C night). Approximately 15-day-old plants, when the third leaves were fully expanded from the whorl (V3 stage), were used for pretreatment. KeYun Pests (https://shop101732681.taobao.com) provided the eggs of *M. separata*. *Mythimna separata* larvae were reared on maize until the third to fifth instar for the collection of OS. Storkbill forceps were used to gently squeeze the caterpillars to provoke regurgitation, and OS were collected on ice with a pipette and immediately centrifuged to obtain supernatant, which was divided into small aliquots before being stored at −80 °C.

### Plant treatments, sample collection, and herbivore bioassays

To study the effects of mechanical wounding- and simulated *M. separata* herbivory-induced priming, the third leaves of maize plants (V3 stage) were pretreated with 10 μl of water or *M. separata* OS at a row of puncture wounds generated by rolling a fabric pattern wheel along the midvein (W+W and W+OS pretreatment, respectively), and these treatments were repeated once a day for another 3 d, unless otherwise indicated; plants without any pretreatments were used as controls. After resting for 3, 7, or 12 d, both control and pretreated plants were treated with W+OS on the fourth leaves by immediately applying 40 μl of *M. separata* OS to four rows of wounds generated by a pattern wheel. Leaf samples were collected 6 or 48 h post-treatment on leaf 4, immediately frozen in liquid nitrogen, and stored at −80 °C until further analysis. All experiments were repeated twice or thrice to ensure data reproducibility except the RNA-seq. The number of replicates for each experiment varied and is indicated in the respective figure caption.

To examine the priming effect of actual *M. separata* feeding, the third maize leaves were infested with *M. separata* larvae (one larva/plant; first instar), and after 4 d of feeding, the insects were removed (pretreatment group). For the control group, no insects were infested on any plants. After another 7 d, the fourth leaves of plants from both control and pretreatment group were infested with *M. separata* larvae (two neonates/plant). The insect masses were recorded after 48 h of feeding. Each group contained 25 replicate maize plants.

To examine the effect of priming on maize resistance to insect herbivory, maize third leaves were pretreated by W+OS for consecutively 4 d (each day 10 μl of *M. separata* OS was applied to one row of wounds generated by a pattern wheel); in the control group, no pretreatment was done. After 3, 7, or 12 d, for each maize plant of both control and pretreatment groups, *M. separata* larvae (two neonates/plant) were enclosed in a clip cage fixed on the fourth leaves and allowed to feed for 48 h before insect masses were recorded. Each group contained 25 replicate maize plants.

### Quantification of jasmonic acid and jasmonic acid–isoleucine conjugate

JA and jasmonic acid–isoleucine conjugate (JA-Ile) were measured according to the method described previously (Wu *et al.*, 2007). In short, 150 mg of frozen leaf powder was extracted with ice-cold ethyl acetate spiked with 20 ng D_6_-JA and 5 ng ^13^C_6_-JA-Ile. After centrifugation at 13 000 *g* for 10 min at 25 °C, supernatants were transferred to fresh 2-ml microfuge tubes. Each pellet was re-extracted with 0.5 ml of ethyl acetate and centrifuged, and the supernatants from each sample were combined. The supernatants were evaporated to dryness on a vacuum concentrator (Eppendorf). The residues were resuspended in 0.5 ml of 70% methanol (v/v) and centrifuged to clarify phases. Following centrifugation, the supernatants were pipetted into glass vials and then analysed by HPLC-MS/MS (LCMS-8040 system, Shimadzu).

### Quantification of benzoxazinoids

Approximately 100 mg of frozen leaf powder was suspended with methanol–H_2_0 (50:50, v/v; containing 0.5% formic acid) in 2 ml microfuge tubes and vortexed vigorously for 10 min. Samples were centrifuged at 13 000 *g* for 15 min and 450 μl of the supernatants was transferred to glass vials for analysis on an HPLC-MS/MS system (LCMS-8040, Shimadzu) according to Gao *et al.* (2019).

### RNA-seq and data analysis

Total RNA was extracted from ground leaf samples using TRIzol reagent (Thermo Fisher Scientific), and the RNA quality, purity, and concentrations were determined using a spectrophotometer (Nano-Drop 2000c, Thermo Fisher Scientific). Sequencing was performed at 5 G depth on a HiSeq2500-PE125 platform (Illumina) and the resulting sequences were trimmed based on quality scores and mapped to the maize A188 reference genome sequence V1. We used HISAT2 (Pertea *et al.*, 2016) to map the transcripts and DESeq2 (Love *et al.*, 2014) to identify differentially regulated genes (DEGs), and genes whose expression levels were at least 2-fold changed with adjusted *P*-values less than 0.05 were selected as DEGs for further analysis. Gene ontology (GO) analysis was done with the updated platform agriGO v2.0 (Tian *et al.*, 2017). Venn diagrams were created using a web-based tool (http://bioinformatics.psb.ugent.be/webtools/Venn/).

### Statistical analysis

Data of herbivore bioassay were analysed with Student’s *t*-test. Analyses on the contents of Bxs and phytohormones were performed using one-way analysis of variance (ANOVA) and significance was determined by *post hoc* test (*P*<0.05). Two-way ANOVA was performed to analyse the effect of time of post-pretreatment resting and priming on Bx contents, and time of resting (3, 7, and 12 d) and priming (pretreatment or not of W+OS on third leaves) were treated as two independent variables. Student’s *t*-test and one-way and two-way ANOVA were performed using SPSS Statistics for Mac OS (IBM Corp., USA; Version 26.0). Principal component analysis was conducted and plotted using the plotPCA in the DESeq2 of the R package (Love *et al.*, 2014). A violin plot was made using the ggplot2 package in R software (Wickham, 2016).

## Results

### Actual and simulated *M. separata* herbivory, but not mechanical wounding, prime maize systemic leaves for increased resistance to *M. separata*

**Fig. 1.**
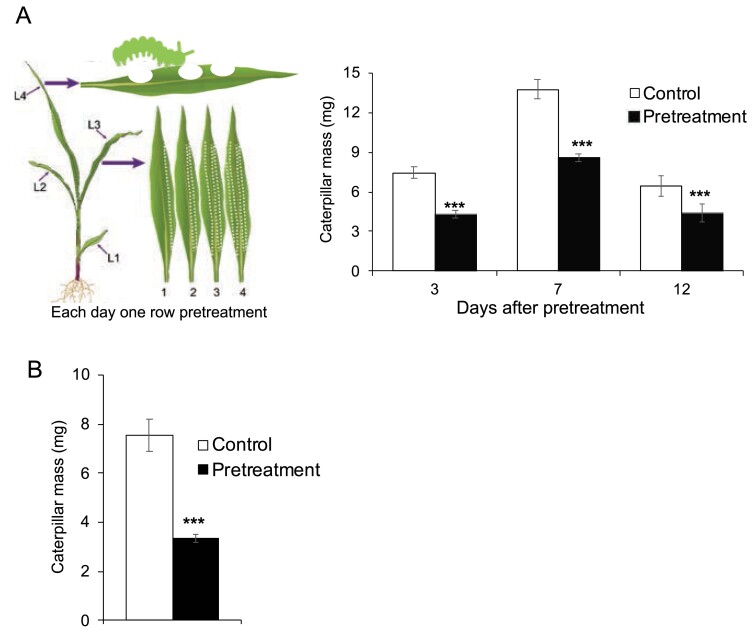
Simulated and actual *M. separata* herbivory primes maize systemic leaves for enhanced resistance. (A) *M. separata* growth on simulated herbivory-pretreated maize. The third leaves (L3) of maize (A188) seedlings were treated with W+OS (one row a day) for four consecutive days (pretreatment group), while in the control group, L3 were untreated. The plants were rested for another 3, 7, or 12 d before *M. separata* larvae were infested on the fourth leaves (L4) (two neonates/plant). The masses of insects after 48 h of feeding were recorded. (B) *M. separata* growth on actual herbivory-pretreated maize. Maize (A188) L3 were infested with *M. separata* larvae (one caterpillar/plant), and after 4 d of feeding, the insects were removed (pretreatment group). In the control group, L3 were untreated. After another 7 d of resting, the L4 were infested with *M. separata* larvae (two neonates/plant), and the insect masses were recorded after 48 h of feeding. Data are mean ±SE; Student’s *t*-test; ***P<0.001; *n*=25.

*Mythimna separata* is one of the major insect pests of maize in Asia. First, we sought to determine whether *M. separata* insect herbivory on maize primes systemic leaves for enhanced resistance. The third leaf of each maize seedling (line A188) was treated with simulated *M. separata* herbivory, by applying the OS of *M. separata* to a row of fresh wounds generated by rolling a pattern wheel along the midvein (wounding plus OS, W+OS). This mode of simulated herbivory was done on four consecutive days on the same local leaves (Fig. 1A). Three, seven, and 12 d (resting times) after the last W+OS treatment, *M. separata* larvae (1 d old and reared on rice seedlings since hatching) were infested on the fourth leaves of these pretreated maize plants. The control maize plants, which were not pretreated, were similarly infested with *M. separata* larvae. The insects were allowed to feed for 48 h before their masses were recorded. Caterpillars fed on the pretreated plants, which had 3, 7, and 12 d of resting, gained only 58, 62, and 69% of the masses of those fed on the control plants (Fig. 1A).

We also confirmed that the caterpillars were 55% smaller on fourth leaves of maize plants whose third leaves were pretreated with actual *M. separata* feeding for 4 d and thereafter rested 7 d, than on the control plants (Fig. 1B). Similar results were also found for the inbred lines B73 and W22 (see Supplementary Fig. S1A): the masses of insects on fourth leaves of B73 and W22, which were pretreated with simulated *M. separata* feeding on third leaves, were 25 and 23% smaller than those on the respective control plants.

**Fig. 2.**
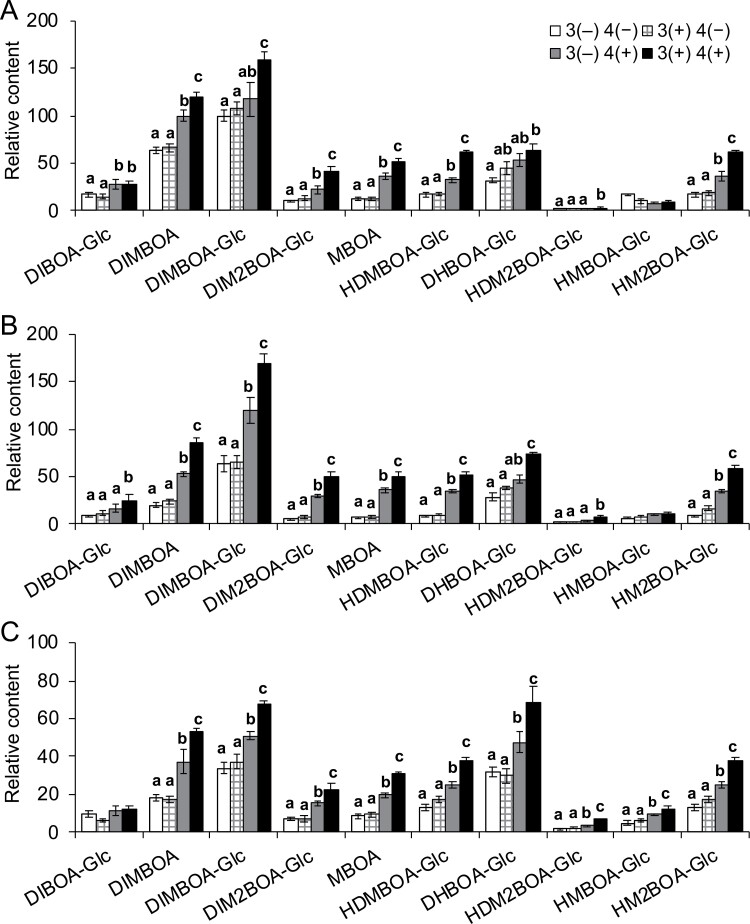
Simulated *M. separata* herbivory primes systemic defenses for at least 12 d. Maize (A188) leaves (third leaves) were pretreated with W+OS for four consecutive days or kept untreated (3(+) and 3(−), respectively). After resting 3 (A), 7 (B), or 12 (C) days, the fourth leaves were treated with W+OS or untreated (indicated as 4(+) and 4(−), respectively). After 48 h, the contents of Bxs in the fourth leaves were analysed. Data are means ±SE (*n*=5). Different lowercase letters indicate significant differences within same compounds determined by one-way ANOVA (*P*<0.05; *post hoc* tests).

Bxs are the major anti-insect metabolites in maize (Ahmad *et al.*, 2011; Meihls *et al.*, 2013). Thus, next we investigated whether simulated *M. separata* herbivory-induced priming in systemic maize leaves is associated with increased contents of Bxs. Third leaves were untreated (for simplicity, named 3(−)) or pretreated with W+OS on four consecutive days (named 3(+)), and after 3, 7, and 12 d of resting, these seedlings’ fourth leaves were untreated (named 4(−)) or treated with W+OS (named 4(+)) to induce Bx accumulation, and these leaves were harvested in another 48 h. In the 3(+) 4(−) and 3(−) 4(−) plants, no differences in Bx contents were found between these two groups, regardless of the resting times (Fig. 2). Thus, W+OS pretreatment on third leaves did not affect the basal levels of Bxs in the fourth leaves. However, the fourth leaves of 3(+) 4(+) plants contained 1.2- to 1.9-fold more Bxs (DIMBOA, DIMBOA-Glc, DIM_2_BOA-Glc, MBOA, HDMBOA-Glc, and HM_2_BOA-Glc) than did the fourth leaves of 3(−) 4(+) plants, which experienced 3 d of resting (Fig. 2A). Similar results were obtained from plants that had experienced 7 and 12 d of resting (Fig. 2B, C). Notably, the seedlings rested for 7 d showed the strongest relative up-regulation of the W+OS-induced Bxs in fourth leaves, compared with those rested for 3 and 12 d (Fig. 2). For instance, W+OS-induced DIMBOA-Glc in the primed plants (3(+) 4(+)) was 34.8, 40.3, and 31.8% more than in the fourth leaves of the non-primed plants (3(−) 4(+)), after 3, 7, and 12 d of resting, respectively. Next, the Bx contents were analysed with two-way ANOVA, in which the resting times (3, 7, and 12 d) and priming/pretreatment (3(+) 4(+) versus 3(−) 4(+)) were considered to be two independent variables. We found that the resting time had a strong impact on levels of Bxs, and for each specific resting time, priming/pretreatment exhibited a significant effect in promoting Bx accumulation (Supplementary Table S1). Thus, both actual and simulated *M. separata* feeding primed the maize seedlings for enhanced herbivore defense, and the herbivory-induced priming in the systemic leaves was associated with increased accumulation of Bxs.

**Fig. 3.**
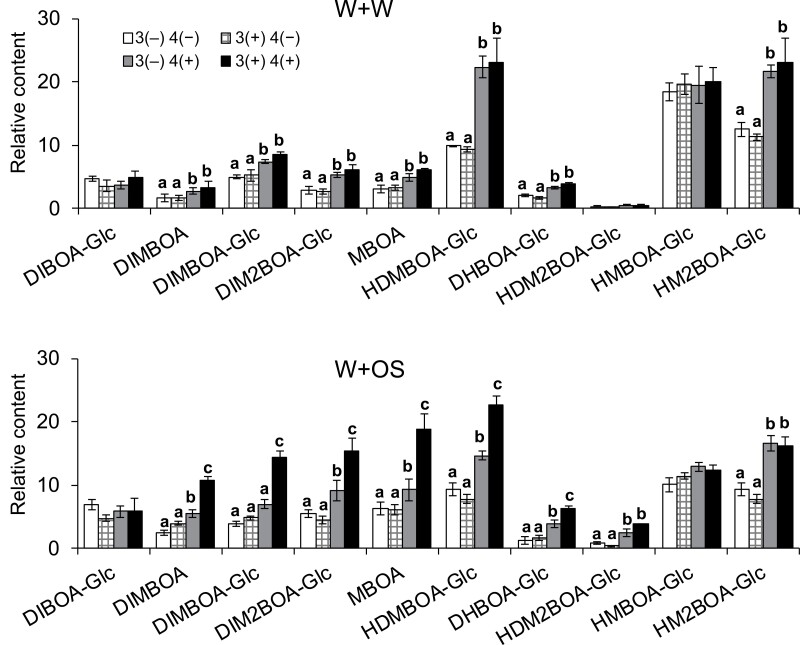
Perception of *M. separata* OS is required for priming of maize systemic leaves. The third maize (A188) leaves were untreated or pre-treated with W+W or W+OS for four consecutive days (indicated as 3(−) and 3(+), respectively). After 7 d of resting, the fourth leaves were untreated or treated with W+OS (indicated as 4(−) and 4(+), respectively). After another 2 d, the contents of Bxs were analysed in the fourth leaves. Data are means ±SE (*n*=5). Different lowercase letters indicate significant differences within same compounds determined by one-way ANOVA (*P*<0.05; *post hoc* tests).

Many insect OS contain various elicitors, such as fatty acid–amino acid conjugates (FACs), and these elicitors can be recognized by plants, inducing insect-specific resistance responses (Wu and Baldwin, 2010). Next, we sought to determine whether insect OS are required for priming the defense of the systemic leaves. In the pretreatment group, the third leaves of maize seedlings were wounded with a pattern wheel, and water was applied to wounds (wounding plus water, W+W). This was done once a day and consecutively for 4 d (similarly to what was done in Fig. 1A). After the plants rested for 7 d, *M. separata* larvae were released to the fourth leaves and their masses were recorded after 2 d of feeding. In the control group, the masses of *M. separata* infested on maize fourth leaves were similarly recorded, except that these maize seedlings were not pretreated. No difference was found between the mass gains of the insects grown on the maize of the pretreatment group and the control group (Supplementary Fig. S1B), indicating that recognition of OS by maize is required for the priming of insect resistance in the systemic leaves. Given that insect feeding- or W+OS-induced priming of systemic leaves was associated with increased Bx contents in the systemic fourth leaves, we expected that mechanical wounding treatment on third leaves may not be able to prime the fourth leaves for enhanced Bx responses to herbivory. The third leaves of maize seedlings were treated with W+W, W+OS, or untreated (control group) once a day for 4 d, and after a resting time of 7 d, W+OS treatment was applied to all the fourth leaves; after another 48 h, these fourth leaves were harvested for determination of Bxs. Compared with those in the control group, the fourth leaves of the maize seedlings from the W+OS pretreatment group again showed enhanced Bx accumulation in response to subsequent W+OS treatment. However, priming was not observed for the W+W pretreatment, as the fourth leaves of the W+W pretreatment group and the control group had similar levels of Bxs after W+OS induction (Fig. 3A, B). These findings indicate that perception of the *M. separata* OS in the local maize leaves is required for priming the systemic leaves for enhanced resistance to insects.

### Duration of simulated herbivory but not extent of damage is required for priming the systemic leaves

**Fig. 4.**
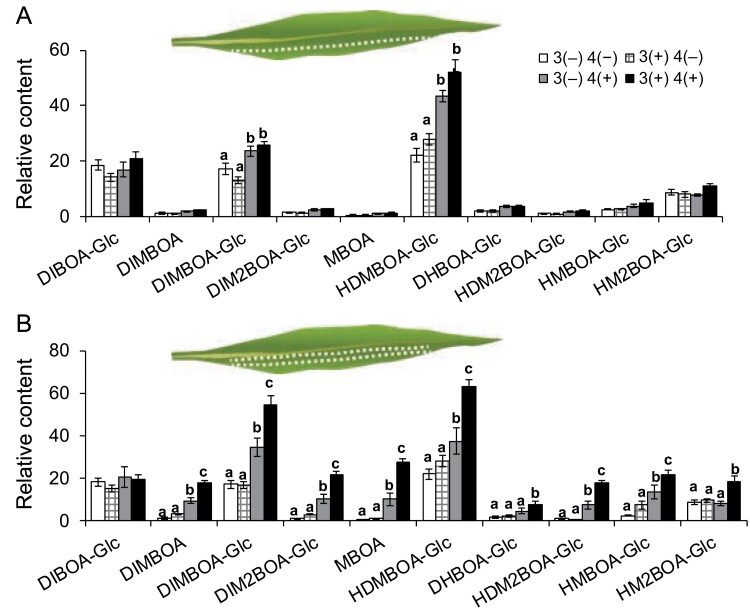
At least two times of simulated *M. separata* treatment are needed to induce priming in maize systemic leaves. Maize (A188) third leaves were untreated or pretreated with W+OS once (A) or once a day on two consecutive days (B) as illustrated, and after 7 d of resting, the fourth leaves were kept untreated or treated with W+OS and harvested after 2 d for quantification of Bxs (3 and 4 depict leaf positions, (+) and (–), respectively, indicate treatment and no treatment. Data are means ±SE (*n*=5). Different lowercase letters indicate significant differences within same compounds determined by one-way ANOVA (*P*<0.05; *post hoc* tests).

Four rolls of W+OS pretreatment primed the systemic fourth leaves, and this led us to ask whether certain damage areas or times of damaging are needed to elicit the priming effect on the fourth leaves. Thus, we generated one roll of wounds in the third leaves of maize seedlings with a pattern wheel and the OS of *M. separata* were immediately applied to wounds (named W+OS once). In another group, maize seedlings were similarly pretreated once a day in two consecutive days (named W+OS twice). After a resting time of 7 d, we treated the fourth leaves of both control (not pretreated at all) and these pre-induced plants with W+OS, and these fourth leaves were harvested in another 2 d for determination of Bx contents. We found that W+OS once did not affect the basal and the subsequently W+OS-induced Bx accumulation in fourth leaves (Fig. 4A); however, W+OS twice highly increased the subsequent W+OS-induced Bxs in fourth leaves, compared with those in the control plants (Fig. 4B).

In order to rule out the possibility that W+OS once was not strong enough to induce priming, in another group of maize seedlings, we generated four rolls of wounds on the third leaves without any time intervals and OS were immediately applied to wounds. After 7 d of resting, fourth leaves were treated with W+OS. However, there were no changes of Bx levels in fourth leaves between the pretreated (3(+) 4(+)) and non-pretreated (3(−) 4(+)) plants except for HDMBOA-Glc (Supplementary Fig. S2). Our findings suggest that herbivore feeding duration but not the extent of damaged area plays an important role in activating priming in the systemic leaves.

### Jasmonic acid and benzoxazinoids are required for herbivore defense priming

**Fig. 5.**
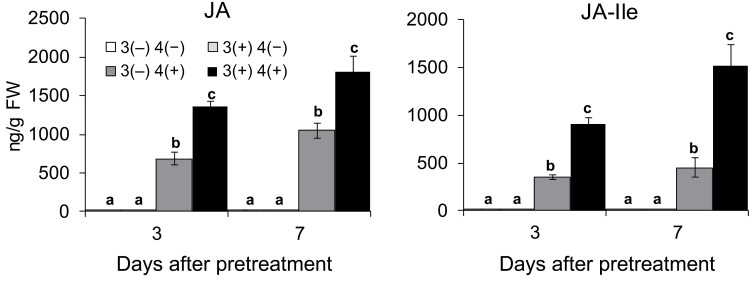
Simulated *M. separata* herbivory primes systemic leaves for increased JA and JA-Ile. Maize (A188) third leaves were untreated or pretreated with W+OS for four consecutive days as shown in Fig. 1A (3(−) and 3(+), respectively). After resting for 3 and 7 d, the fourth leaves were untreated or treated with W+OS (4(−) and 4(+), respectively) and after another 1 h, samples of fourth leaves were collected for analysing JA and JA-Ile contents (means ±SE, *n*=7). Different lowercase letters indicate significant differences within each time point determined by one-way ANOVA (*P*<0.05; *post hoc* tests).

Phytohormones, especially JA, play important roles in regulating defensive metabolites (De Geyter *et al.*, 2012; Howe *et al.*, 2018). Therefore, we speculated that simulated herbivory on third leaves could prime the fourth leaves for enhanced JA response to subsequent W+OS treatment. Maize seedlings (third leaves) were pretreated with W+OS for consecutive 4 d. Three or seven days after pretreatment, the systemic fourth leaves of the plants from the pretreated group and control group were treated with W+OS and samples were harvested after 1 h. It was found that the levels of JA in the fourth leaves were 50% and 43% higher in the pretreatment group than in the control group, after 3 and 7 d of resting, respectively (Fig. 5). Similar results were found for the actual functional jasmonate, jasmonic acid–isoleucine conjugate (JA-Ile) (Fig. 5).

**Fig. 6.**
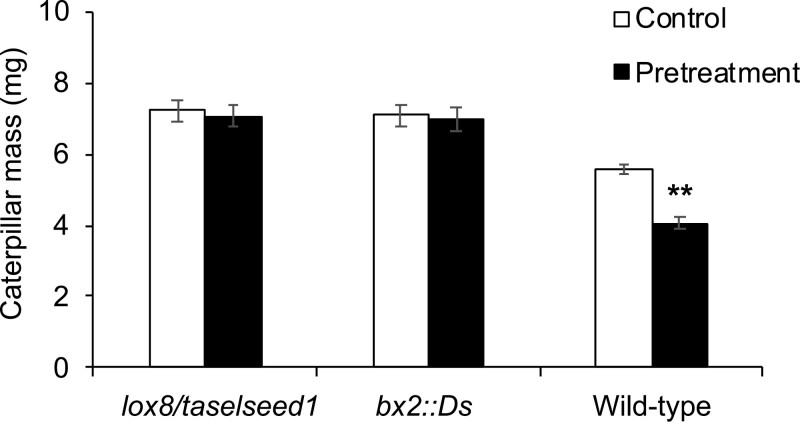
Priming-enhanced maize resistance to *M. separata* is JA- and Bx-dependent. The third leave of wild-type maize (W22) and mutants *bx2::Ds* and *lox8/taselseed1* were untreated (control) or pretreated with W+OS for consecutively 4 d (pretreatment), as shown in Fig. 1A. After 7 d of resting, the fourth leaves were infested with *M. separata* larvae and their masses were recorded 2 d after infestation. Data are mean ±SE; Student’s *t*-test; **P<0.001; *n*=25.

To further investigate whether the primed defenses in the the systemic leaves depend on the JA signaling and defensive Bx metabolites, we employed the *lox8/tasselseed1* mutant, which is impaired in JA biosynthesis, and the *bx2::Ds* mutant, which lacks Bxs. For the WT maize line W22, which is the genetic background of these two mutants, and the *lox8/tasselseed1* and *bx2::Ds* mutant, we pretreated the third leaves with 4 d of W+OS or untreated (controls) and after 7 d of resting, the fourth leaves were infested with *M. separata* insects for 48 h. On the pretreated W22, *M. separata* grew 27% smaller than the insects grown on the non-pretreated control W22 group (Fig. 6). Importantly, on the *lox8/tasselseed1* and *bx2::Ds* mutant plants, the caterpillars exhibited very similar masses on the control and pretreated plants (Fig. 6). Notably, insects grown on *lox8/tasselseed1* and *bx2::Ds* were always larger than those on the W22 plants, under the pretreatment or control conditions (Fig. 6), confirming the important role of JA signaling and Bx defensive metabolites. We confirmed that the *lox8/tasselseed1* and *bx2* mutant plants accumulated very little Bxs (Supplementary Fig. S3A, B), and W+OS pretreatment on third leaves did not have a priming effect on the subsequent W+OS-induced Bxs in fourth leaves (Supplementary Fig. S3).

Thus, W+OS-induced priming in fourth leaves against subsequent insect attack requires JA signaling and Bx accumulation.

### Priming enhances transcriptional regulation of various defense-related genes in systemic leaves in response to W+OS

To gain insight into the underlying molecular mechanism of defense priming, we performed a global gene expression analysis on the maize seedlings. The W+OS-pretreated and untreated seedlings (on the third leaves) were allowed to rest for 7 d, and their fourth leaves were treated with W+OS (3(+) 4(+) and 3(−) 4(+), respectively) or untreated (3(+) 4(−) and 3(−) 4(−), respectively). After 6 h, the fourth leaves of these four groups were sampled for RNA-seq analysis.

Principle component analysis (PCA) indicated little variations among the three biological replicates (Supplementary Fig. S4), and the first component PC1 accounted for 90% of the variations among samples and clearly clustered the samples of the 3(−) 4(−), 3(+) 4(−), 3(−) 4(+), and 3(−) 4(+) groups. Compared with the 3(−) 4(+) samples, samples of the 3(+) 4(+) group clustered furthest from the untreated control, 3(−) 4(−), indicating that pretreatment followed by subsequent W+OS treatment in fourth leaves induced the greatest changes in the systemic fourth leaves (Supplementary Fig. S4).

**Fig. 7.**
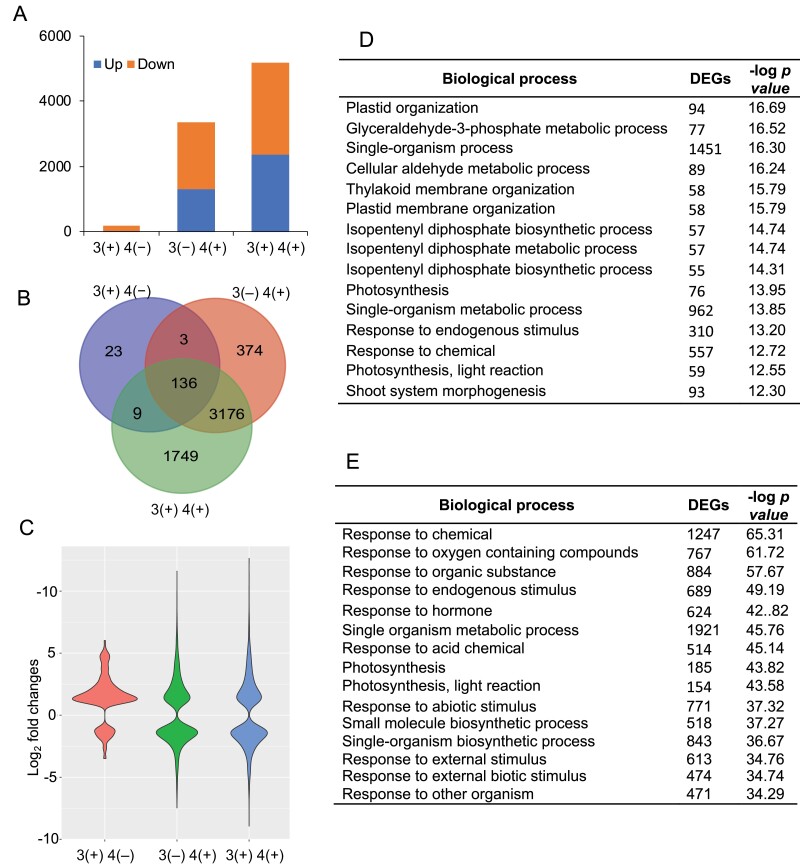
Primed maize leaves exhibit large transcriptional changes in response to simulated herbivory. Maize (A188) third leaves were untreated or pretreated with W+OS for four consecutive days as shown in Fig. 1A (3(−) and 3(+), respectively). After 7 d of resting, the fourth leaves were untreated or treated with W+OS (4(−) and 4(+), respectively) and after another 6 h, samples of fourth leaves were collected for RNA-seq analysis. (A) Numbers of up- and down-regulated genes in 3(+) 4(−), 3 (–) 4(+), and 3(+) 4(+), compared with 3(−) 4(−) samples. (B) Venn diagram depicting the specifically and commonly regulated DEGs. (C) Violin plot depicting the quantitative expression of all DEGs in 3(+) 4(−), 3(−) 4(+), and 3(+) 4(+) samples. (D, E) Enriched GO terms (biological process) from the DEGs unique for the 3(+) 4(+) group (D) and from the genes that are common for both 3(−) 4(+) and 3(+) 4(+) groups but are further up- or down-regulated in the 3(+) 4(+) group (E).

Using the transcriptome data from the fourth leaves of the 3(−) 4(−) (control) group as the reference, DEGs were inferred from all the other groups. In total, 5470 DEGs were identified (Supplementary Table S2). The fourth leaves of the 3(+) 4(−) group, which were only pretreated with W+OS, had only 171 DEGs (35 up- and 136 down-regulated). After W+OS treatment, the primed fourth leaves (3(+) 4(+)) showed, respectively, 2322 and 2748 up- and down-regulated DEGs, while in the non-primed group (3(−) 4(+)) there were 1678 up- and 2011 down-regulated genes in the fourth leaves (Supplementary Table S2; Fig. 7A). Venn diagram analysis indicated that the fourth leaves of the 3(−) 4(+) and 3(+) 4(+) groups have 3312 common DEGs (Supplementary Table S3; Fig. 7B); among the common genes, 2542 genes (76.7%) exhibited at least 10% further increased (2276 genes) or decreased (266 genes) transcript levels in the 3(+) 4(+) than in the 3(−) 4(+) group and 463 genes (14%) were found to have at least 50% further increased (456 genes) or decreased (7 genes) levels in the 3(+) 4(+) than in the 3(−) 4(+) group. Importantly, in the 3(+) 4(+) group 1749 genes were specifically up- or down-regulated, while in the non-primed 3(−) 4(+) group there were only 374 specifically regulated genes (Supplementary Table S3; Fig. 7B). Consistently, violin plot analysis showed that many genes in the 3(+) 4(+) samples were more strongly regulated than in the 3(−) 4(+) samples (Fig. 7C). These data suggest that pretreatment on third leaves enabled the fourth leaves to respond with transcriptional changes of a large number of genes, including many unique genes.

Next, gene ontology (GO) analysis was used to gain insight into the function of priming-related DEGs. The enriched biological processes from the uniquely regulated genes in the fourth leaves of the 3(+) 4(+) group included ‘plastid organization’, ‘glyceraldehyde-3-phosphate metabolic process’, ‘single-organism process’, and ‘cellular aldehyde metabolic process’ (Supplementary Table S4; Fig. 7D). Furthermore, GO terms enriched from the genes that were common in the 3(−) 4(+) and 3(+) 4(+) groups and were further promoted/suppressed in the 3(+) 4(+) group included ‘response to chemical’, ‘response to oxygen containing compounds’, ‘response to organic substance’, and ‘response to endogenous stimulus’ (Fig. 7E). The unique DEGs regulated in the 3(−) 4(+) group were not enriched in any GO terms.

Given that the accumulation of JA and Bxs was clearly regulated by priming, we specifically inspected the expression levels of JA and BX biosynthetic genes in different groups. Strikingly, among JA biosynthetic genes and catabolic genes, only *LOX3* and *bngle1371*, a JA biosynthetic and a catabolic gene, respectively, were slightly but significantly increased in the fourth leaves of the 3(+) 4(+) group compared with the 3(−) 4(+) group (Supplementary Fig. S5). Similarly, among the 14 BX biosynthetic genes, only four, *BX10*, *BX11*, *BX13*, and *BX14*, were found to show priming-enhanced expression levels (i.e. greater levels in the fourth leaves of the 3(+) 4(+) group than in the 3(−) 4(+) group), while the rest of the BX genes seemed not to be involved in priming (Supplementary Fig. S6).

## Discussion

In this study, we investigated the herbivory-induced priming of defense responses in the systemic leaves of maize seedlings. Our results demonstrate that actual or simulated *M. separata* herbivory induced priming in the unattacked systemic leaves, resulting in stronger and faster defenses upon subsequent caterpillar attack. The primed state could persist for at least 12 d in the systemic leaves, and the increased defense induced by herbivory on the systemic leaves was dependent on the JA pathway and Bx biosynthesis.

Previous studies have documented defense priming against insect herbivory, and the priming was induced by HIPVs, oviposition, β-amino-butyric acid, or cytokinin (Engelberth *et al.*, 2004; Conrath *et al.*, 2006; Frost *et al.*, 2007; Dervinis *et al.*, 2010). For example, indole primed the neighboring maize plants for enhanced release of herbivory-induced defense volatiles as well as increased expression of early defense signaling genes (Erb *et al.*, 2015; Ye *et al.*, 2019). Similarly, HIPVs released from *M. separata*-infested maize increased the resistance of downwind maize to insect herbivory, and the primed maize exhibited highly increased transcript levels of Bowman–Birk type trypsin inhibitor, compared with the maize plants receiving volatiles from the untreated maize (Ali *et al.*, 2013). However, HIPV-induced defense priming seems to be species-specific. For example, exposure of the wild tobacco *N. attenuata* to volatiles released from simulated *M. sexta* feeding-induced *N. attenuata* did not result in different profiles of secondary metabolites or JA from those exposed to volatile from untreated controls (Paschold *et al.*, 2006). In addition to HIPVs, the vasculature also conveys herbivory-induced systemic signals, which likely play essential roles in priming the defense of systemic tissues. Erb *et al.* (2008) showed that belowground herbivory by *D. v. virgifera* induced resistance to the caterpillar *S. littoralis* in maize leaves, and these leaves exhibited priming for elevated chlorogenic acid after subsequent *S. littoralis* feeding. Previously, it was found that simulated herbivory in maize had a strong effect on responses of systemic leaves, including increased JA levels and accumulation of Bxs (Malook *et al.*, 2019). In our experiments, simulated herbivory-induced accumulation of Bxs in the systemic leaves could be ruled out, as quantification of Bxs indicated that the 3(−) 4(−) and 3(+) 4(−) leaves had the same concentrations of Bxs (Fig. 2); that is the enhanced defense in the fourth leaves was due to priming, but not because pretreatment on third leaves increased the contents of Bxs in fourth leaves (Fig. 2). A188, B73, and W22 all showed priming responses (Supplementary Fig. S1), suggesting that priming is a general trait of the maize defense system. However, Maag *et al.* (2016) used *S. littoralis* caterpillars to feed on local maize leaves for 24 h and 48 h, but they did not find any priming effect in the systemic leaves. The discrepancy between our study and Maag *et al.* (2016) may be due to different insect species: the generalist *S. littoralis* may be able to suppress systemic priming, while the specialist *M. separata* cannot. Another possibility is that *S. littoralis* insects used by Maag *et al.* (2016) were too small to make enough damage to induce priming in the systemic leaves, as we found that there was no systemic priming if simulated herbivory was performed by applying *M. separata* OS to only one row of mechanical wounds (Fig. 4).

We show that systemic defense priming is dependent on perception of certain elicitors in the OS of *M. separata* (Fig. 3). Previously it has been shown that *M. separata* OS are rich in several types of FACs (Qi *et al.*, 2016), which are known to be potent elicitors in various insect OS (Wu and Baldwin, 2010). Similarly, in *N. attenuata*, simulated *M. sexta* herbivory (applying OS of *M. sexta* to wounds) but not mechanical wounding, activated salicylic acid-induced protein kinase and JA biosynthesis in systemic leaves (Hettenhausen *et al.*, 2014). Thus, it is conceivable that maize perception of FACs in the *M. separata* OS is required for inducing the mobile priming agents. Even though the nature of mobile priming agents that promote systemic leaves into a primed state remains unclear, it is likely that these agents are a part of the herbivory-induced mobile systemic signals, which are possibly associated with Ca^2+^, reactive oxygen species, and ion channels (Hilleary and Gilroy, 2018; Kumari *et al.*, 2019). It would be interesting to study whether maize mutants impaired in these signaling pathways have phenotypes of compromised defense priming in the systemic leaves.

In this study, we show that applying *M. separata* OS to one or even four rows of wounds in third leaves at once did not prime the systemic fourth leaves; in contrast, applying *M. separata* OS to one row of wounds once a day on two consecutive days successfully induced priming (Fig. 4). This was consistent with the findings that Arabidopsis that suffered from repetitive heat, cold, or salt stress was primed to have increased resistance to *Pst* DC3000, while long-term exposure to heat, cold, or salt did not prime the plants (Singh *et al.*, 2014). Repeated treatment of simulated *Manduca sexta* herbivory on the wild tobacco *N. attenuata* plants elicited more rapid JA bursts and discrete increase in basal levels of JA and JA-Ile (Stork *et al.*, 2009). Similarly, Baldwin and Schmelz (1996) showed that prior elicitation of the *Nicotiana sylvestris* plants twice with methyl jasmonate primed the plants for more rapid accumulation of nicotine upon a third treatment with methyl jasmonate, compared with naïve plants or plants that were pretreated only once. Although priming enables plants to gain higher fitness than non-primed ones in the presence of stresses, priming likely incurs costs (van Hulten *et al.*, 2006); thus, being able to sense insect feeding, which includes repetitive wounding and sensing insect-specific elicitors, is necessary for minimizing the costs. It is unclear how repetitive W+OS primes maize systemic leaves.

We analysed the effect of priming on transcriptome changes (Fig. 7). The primed 3(+) 4(+) group exhibited many specifically induced/repressed genes compared with the non-primed 3(−) 4(+) (Fig. 7B). Detailed inspection of the transcriptome data indicated that (i) 34.5% of the total DEGs in the 3(+) 4(+) group were specifically regulated by priming; namely, these genes were regulated only if third leaves were pretreated with W+OS; (ii) 374 unique genes (10.1% of all DEGs in the 3(−) 4(+) group) were no longer regulated, when third leaves were pretreated, in other words, if fourth leaves were primed; and (iii) 68.9% (2542 genes) of all DEGs in the 3(−) 4(+) group showed further up- or down-regulation (at least 10%), if third leaves were pretreated. We hypothesize that in addition to the contribution of the 1749 specifically regulated genes in the fourth leaves of the 3(+) 4(+) group, the 2542 genes that were further induced/suppressed by priming could also play a role in priming-induced defense responses. How priming enables the systemic fourth leaves to mount strong defenses against *M. separata*, including strongly altered transcriptome and enhanced accumulation of defensive Bxs, remains unclear. Epigenetic changes have been detected in stress-treated plants (Jaskiewicz *et al.*, 2011; Luna *et al.*, 2012). Stresses, such as phosphate starvation or pathogen infection, lead to genome-wide methylome reconfigurations, which are often associated with transcriptome changes (Dowen *et al.*, 2012; Secco *et al.*, 2015; Yong-Villalobos *et al.*, 2015). In our maize–*M. separata* interaction system, the involvement of maize epigenetic changes after actual/simulated herbivory seems to be very likely.

The primed resistance was abolished in the JA biosynthesis *lox8* mutants and Bx biosynthesis *bx2* mutants (Supplementary Fig. S3). Therefore, it is very likely that certain priming-associated responses are upstream of JA biosynthesis, as indicated by the finding that primed plants had much higher concentrations of W+OS-induced JA than did the non-primed plants. A similar finding was that touching Arabidopsis leaves repetitively increased the resistance of these leaves to the fungus *Botrytis cinerea* and cabbage looper (*Trichoplusia ni*), while touch-induced priming was not detected in the JA-deficient *aos* mutants (Goodspeed *et al.*, 2012). Pretreatment by simulated herbivory strongly primed maize for enhanced levels of JA (Fig. 5) and Bxs (Fig. 2), but detailed inspection of the JA biosynthetic and catabolic genes and Bx biosynthetic genes indicated that only a few genes fitted into the transcriptional pattern of priming (Supplementary Figs S5, S6). Thus, it is possible that in addition to transcriptional regulation, post-transcriptional regulation of JA and Bx biosynthetic and catabolic genes may play an important role in priming. Given that we did not examine the expression levels of JA and Bx biosynthetic genes at multiple times (only at 6 h), there is another possibility, that priming affects the expression of JA and Bx biosynthetic genes at earlier or later times than at 6 h. The mechanism of defense priming deserves further study.

Taken together, in this study we show that maize is able to sense repetitively treated simulated herbivory and deploy priming in the systemic leaves, thus enabling these leaves to respond to subsequent insect feeding with highly increased JA and defensive Bx metabolites. Our analysis reveals that priming promotes transcriptional activation in the systemic leaves. These results provide new insight into priming, a process that is important for maize defense against insects. Further studies on the systemic and priming-associated signals will further uncover the mechanism underlying priming, which could facilitate breeding of maize lines with enhanced resistance to insects.

## Supplementary data

The following supplementary data are available at *JXB* online.

Fig. S1. Simulated *M. separata* herbivory-induced priming is conserved in different maize lines and requires perception of OS.

Fig. S2. More than one time of simulated *M. separata* treatment is required to induce priming in maize systemic leaves.

Fig. S3. *Bx2* and *lox8/taselseed1* mutants do not exhibit priming-induced elevation of Bxs.

Fig. S4. Overview of RNA-seq data from maize fourth leaves after different treatments.

Fig. S5. JA biosynthesis and catabolism genes in 3(−) 4(+) and 3(+) 4(+) groups do not have different transcript levels.

Fig. S6. Most Bx biosynthesis genes in 3(−) 4(+) and 3(+) 4(+) groups do not have different transcript levels.

Table S1. ANOVA table from two-way ANOVA considering resting time and priming as independent variables.

Table S2. All DEGs in maize systemic leaves of 3(+) 4(−), 3(−) 4(+), and 3(+) 4(+) groups.

Table S3. Common and specific DEGs in maize systemic fourth leaves of 3(+) 4(−), 3(−) 4(+), and 3(+) 4(+) groups.

Table S4. Gene ontology enrichment from the specifically and commonly regulated DEGs in maize systemic fourth leaves of 3(+) 4(−), 3(−) 4(+), and 3(+) 4(+) groups.

erab083_suppl_Supplementary_Figures_S1-S6

erab083_suppl_Supplementary_Table_S1

erab083_suppl_Supplementary_Table_S2

erab083_suppl_Supplementary_Table_S3

erab083_suppl_Supplementary_Table_S4

## Data Availability

The raw data were deposited in the National Genomics Data Center (https://bigd.big.ac.cn) and available under the BioProject ID (PRJCA003876).
