# Supplementary material for: Mythimna separata herbivory primes maize resistance in systemic leaves
Source: J Exp Bot. 2021 Feb 27;72(10):3792–805. doi: 10.1093/jxb/erab083 (PMC8096606; doi:10.1093/jxb/erab083)
Supplement: erab083_suppl_Supplementary_Figures_S1-S6 [file erab083_suppl_supplementary_figures_s1-s6.pdf]

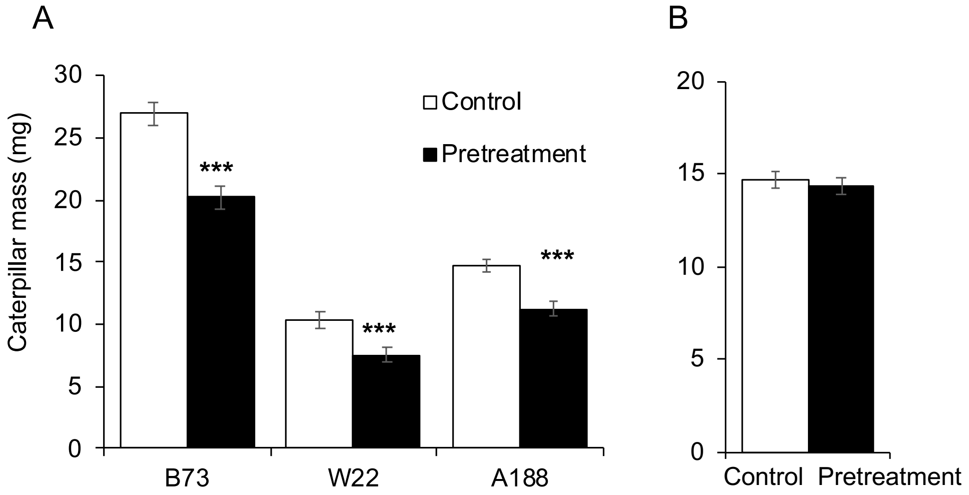

Fig. S1. Simulated *M. separata* herbivory-induced priming is conserved in different maize lines and requires perception of OS.

(A) *M. separata* growth on simulated herbivory-pretreated maize lines B73, W22, and A188. The third leaves (L3) of maize seedings were treated with W+OS (one row a day) for four consecutive days (pretreatment group), while in the control group, L3 were untreated. The plants were rested for another 7 days, before *M. separata* larvae were infested on the fourth leaves (L4) (one caterpillar/plant). The masses of insects after 48 h of feeding were recorded. (B) *M. separata* growths on control and wounding-pretreated A188 seedlings. Maize (A188) L3 were treated with W+W (one row a day) for four consecutive days (pretreatment group), while in the control group, L3 were untreated. After 7 days of resting, the fourth leaves were infested with *M. separata* larvae (two neonates/plant), and the insect masses were recorded after 48 h of feeding (no significant differences were detected between control and pretreatment group). Data are mean ± SE; Student's *t*-test; ***, $P < 0.001$; n = 25.

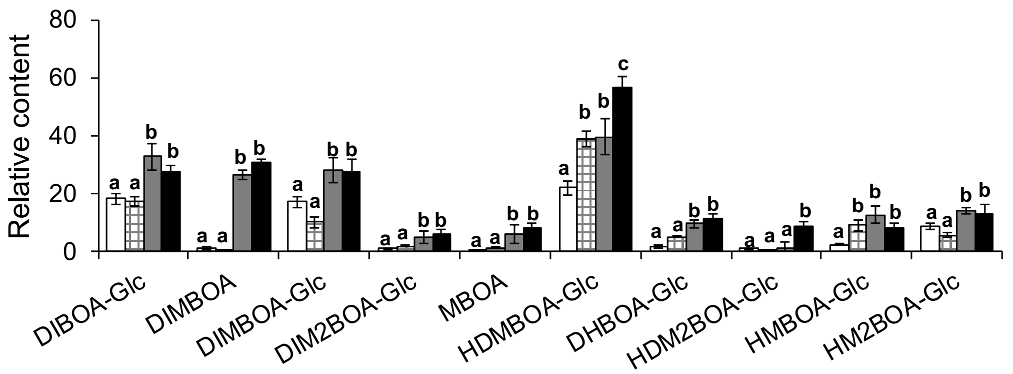

Fig. S2. More than one time of simulated *M. separata* treatment is required to induce priming in maize systemic leaves.

Maize third leaves were untreated or pretreated with W+OS as illustrated in Figure 1A, except that the treatment was performed without any time intervals. After 7 days of resting, the fourth leaves were kept untreated or treated with W+OS and harvested after 2 days for quantification of Bxs. In legends, 3 and 4 depict leaf positions, (+) and (–) respectively indicate treatment and no treatment. Data are means ± SE (n = 5). Different small letters indicate significant differences within same compounds determined by one-way ANOVA ($P < 0.05$; post-hoc tests).

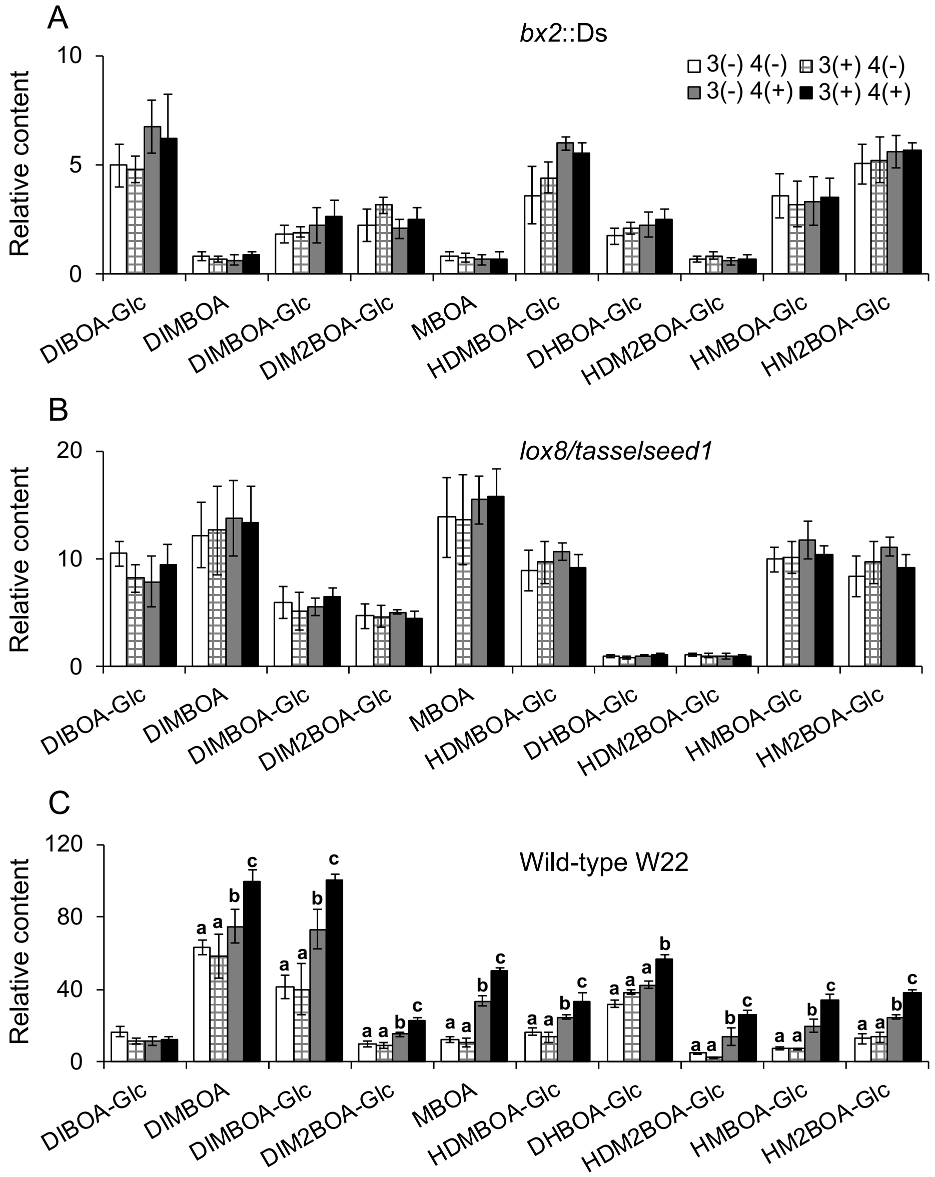

Fig. S3. *Bx2* and *lox8/taselseed1* mutants do not exhibit priming-induced elevation of Bxs.

The third leave of wild-type maize (W22) and mutants *bx2::Ds* and *lox8/taselseed1* were untreated (control) or pretreated with W+OS for consecutively 4 days (pretreatment), as shown in Figure 1A. After resting of 7 days, the fourth leaves were untreated or treated with W+OS (indicated as 4(–) and 4(+) respectively). After another 2 days, the contents of Bxs were analysed in fourth leaves. Data are means ± SE (n = 5). Different small letters indicate significant differences within same compounds determined by one-way ANOVA ($P < 0.05$; post-hoc tests).

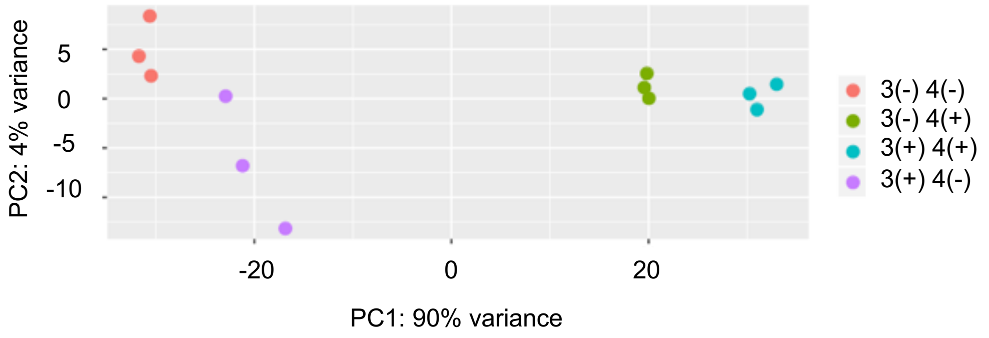

Fig. S4. Overview of RNA-seq data from maize fourth leaves after different treatments.

Maize (A188) third leaves were untreated or pretreated with W+OS for 4 consecutive days (3(–) and 3(+), respectively). After 7 days of resting, the fourth leaves were untreated or treated with W+OS (4(–) and 4(+), respectively). After another 6 h, samples of fourth leaves were collected for RNA-seq analysis. PLS-DA plot was drawn using count data table of 3(–)4(–), 3(+)4(–), 3(–)4(+), and 3(+)4(+) groups.

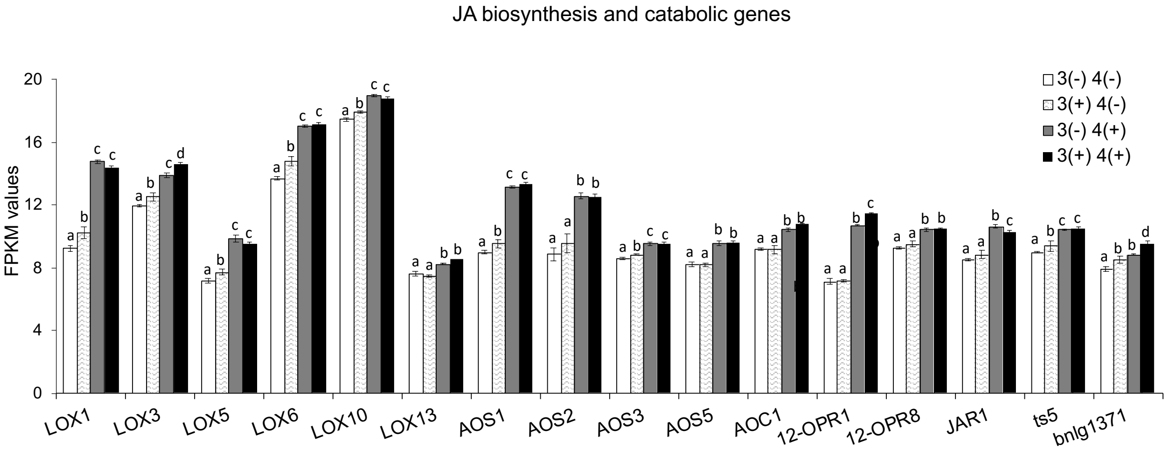

Fig. S5. JA biosynthesis and catabolism genes in 3(–) 4(+) and 3(+) 4(+) group do not have different transcript levels.

Maize (A188) third leaves were untreated or pretreated with W+OS for 4 consecutive days (3(–) and 3(+), respectively). After 7 days of resting, the fourth leaves were untreated or treated with W+OS (4(–) and 4(+), respectively). After another 6 h, samples of fourth leaves were collected for analyzing global transcriptomic changes. The relative transcript levels of genes involved in JA biosynthesis and catabolism were retrieved from the RNA-seq data (n = 3). Different small letters indicate significant differences within same gene determined by one-way ANOVA ($P < 0.05$; post-hoc tests).

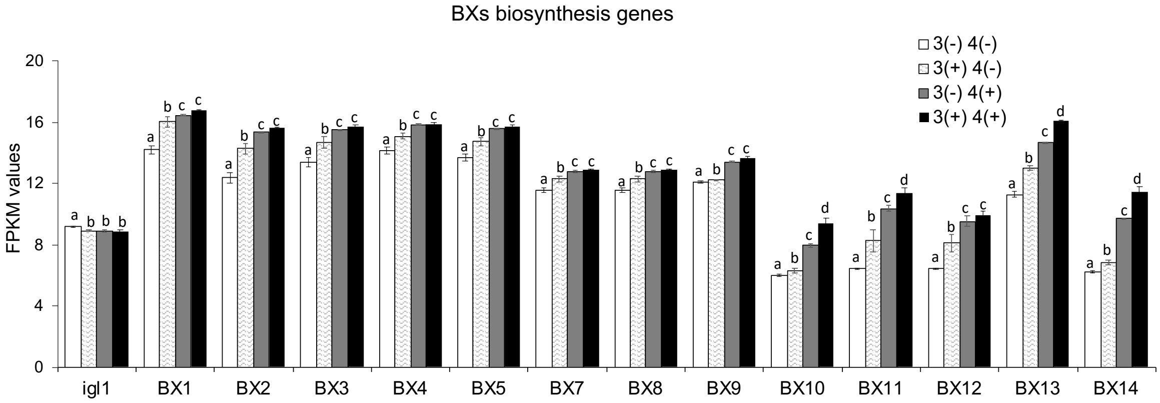

Fig. S6. Most Bx biosynthesis genes in 3(–) 4(+) and 3(+) 4(+) group do not have different transcript levels.

Maize (A188) third leaves were untreated or pretreated with W+OS for 4 consecutive days (3(–) and 3(+), respectively). After 7 days of resting, the fourth leaves were untreated or treated with W+OS (4(–) and 4(+), respectively). After another 6 h, samples of fourth leaves were collected for analyzing global transcriptomic changes. The relative transcript levels of genes involved in Bx biosynthesis were retrieved from the RNA-seq data (n = 3). Different small letters indicate significant differences within same gene determined by one-way ANOVA ($P < 0.05$; post-hoc tests).
